# The Existence-Relatedness-Growth Theory for Job Satisfaction and Motivation of Greek National Healthcare Service Employees in the Context of Severe Financial Constraints

**DOI:** 10.7759/cureus.62738

**Published:** 2024-06-19

**Authors:** Emmanouil Koutalas, Evangelos Kostares, Ioannis Antonakos, Eva Paraskevadaki, Theodora Psaltopoulou, Elena Riza, Konstantinos Tsiamis, Athanasios Tsakris, Maria Kantzanou

**Affiliations:** 1 Microbiology, National and Kapodistrian University of Athens School of Medicine, Athens, GRC; 2 Radiology, National and Kapodistrian University of Athens School of Medicine, Athens, GRC; 3 Clinical Therapeutics, National and Kapodistrian University of Athens School of Medicine, Athens, GRC; 4 Epidemiology and Public Health, National and Kapodistrian University of Athens School of Medicine, Athens, GRC; 5 Public and One Health, University of Thessaly, Thessaly, GRC

**Keywords:** greek national healthcare service, financial constraints, job satisfaction, erg theory, motivation factors, interdisciplinary workflow

## Abstract

The hospital environment is often quite complicated due to interdisciplinary workflow procedures and multitasking staff, which are exacerbated during periods of economic crisis. This study aimed to examine the motivation and job satisfaction factors of Greek National Healthcare Service (NHS) employees in relation to the Existence-Relatedness-Growth (ERG) theory of motivation during a period of severe financial constraints. A cross-sectional study was conducted in three public hospitals in Greece from 2018 to 2019, utilizing a survey tool to measure the factors of motivation and job satisfaction among Greek NHS employees. The study also aimed to identify the most relevant motivational theory applicable to the complex Greek hospital environment. Exploratory factor analysis (EFA) was employed to extract the structural factors of the survey tool, and analysis of variance (ANOVA) was used to identify statistical differences between the means of three or more independent groups. A sample of 363 Greek NHS employees participated in this study. Statistically significant differences were detected between hospital units and job satisfaction factors, as well as between the functions of hospital clusters and job positions. Specifically, managerial staff presented higher levels of job satisfaction, while nursing staff had the lowest scores in terms of psychological contracts when compared to medical and administrative staff. This study demonstrated that job satisfaction in Greek public hospitals, in a context of severe financial constraints, was mainly driven by strong interpersonal connections and employee trust in management, despite significant cuts in salaries, staff numbers, and hospital budgets.

## Introduction

The Greek National Healthcare Service (NHS) has faced numerous challenges since its inception, including inefficiencies and distortions in corporate governance, human resource management, and resource allocation [1]. The governance of the Greek NHS, particularly in management and personnel recruitment, remains centralized and subject to governmental changes. This centralization limits the autonomy of hospital managers and boards, hindering their ability to effectively develop and implement business plans [2]. Often, managers are appointed based on political criteria rather than managerial skills and professional experience, resulting in outdated leadership practices and poor organizational culture and motivation policies [3].

The Greek NHS was severely impacted by the financial crisis that followed the global financial crisis of 2008. Austerity measures and restructuring policies led to salary cuts of up to 40%, staff reductions between 10% and 40%, and a 30% decrease in hospital budgets [4]. These measures failed to address the underlying structural deficiencies, exacerbating the negative impact on employees’ working conditions [5-7]. Job satisfaction, influenced by wages, working conditions, job security, HR policies, and organizational environment, was directly affected by these adverse economic conditions. The financial constraints persisted even after the official end of the crisis period, continuing to affect the NHS post-pandemic, with a severe shortage of nurses and specialists such as radiologists, anesthesiologists, and intensive care doctors. Studies have shown that the economic crisis and high unemployment significantly impacted job security satisfaction and affective commitment, shifting the focus to extrinsic satisfaction factors [7-10].

Motivation theories, particularly those of Maslow and Herzberg, have been foundational in studying the motivation and job satisfaction of healthcare professionals in Greece [11-13]. Maslow’s hierarchy of needs suggests that individuals move to higher levels of needs once lower levels are satisfied. Herzberg’s two-factor theory distinguishes between motivators (e.g., achievement, recognition) and hygiene factors (e.g., salary, working conditions), which influence job satisfaction [10-13]. However, evidence suggests that during crises, motivation and job satisfaction do not always follow these straightforward models. Public sector employees are more motivated and satisfied than private sector employees, even when faced with resource shortages and salary cuts. This is attributed to intrinsic rewards and recognition practices, as well as a sense of duty and values related to the social role of public hospital services [13]. Alderfer’s Existence-Relatedness-Growth (ERG) theory, developed by Clayton Alderfer, categorizes human needs into three core groups: existence, relatedness, and growth. Existence needs include basic material and physiological requirements such as food, water, and safe working conditions. Relatedness needs pertain to the desire for interpersonal relationships and social interactions with family, friends, and colleagues. Growth needs involve the intrinsic desire for personal development, creativity, and achieving one’s full potential. Unlike Maslow’s hierarchy, ERG theory allows for these needs to be pursued simultaneously and recognizes that individuals can regress to lower-level needs when higher-level needs are not met [14,15].

There is limited evidence on the impact of fiscal reforms on the motivation and job satisfaction of Greek NHS employees during the era of partial exit from Greek memorandums. This study aimed to investigate the drivers of employee satisfaction in the Greek NHS under severe financial constraints. It also examined how satisfaction levels varied within different hospital units that underwent various reforms since 2008. Additionally, the study explored the relationship between psychological contract breach, trust, engagement, and professional relations across different demographics and job characteristics.

## Materials and methods

Study design and data collection

This is a cross-sectional observational study conducted during the third and fourth quarters of 2018 in the Greek hospital cluster serving the area of northeast Attica, which was subjected to memorandum reforms from 2010 to 2018-2019. The hospital cluster comprised three units: Sismanoglio General Hospital with 1,050 employees, Amalia Fleming Hospital with 350 employees, and Paidon Pentelis Hospital with 400 employees. To ensure broad participation, the Hospital Administration sent a detailed email to all departments within the hospital units. This email outlined the study's aims, emphasized the voluntary and anonymous nature of participation, and assured staff of the secure handling of all collected data. Collection boxes for the questionnaires were strategically placed in accessible locations throughout each hospital unit. Employees were invited to voluntarily complete the questionnaires and return them in sealed envelopes to these collection boxes. The Office of Quality and Education of the Sismanoglio Hospital Unit collected the completed questionnaires, transferred the data into digital form, and forwarded the data to the researcher. They then digitized the data and forwarded it to the researcher for analysis. The self-reported questionnaire, accompanied by a letter reiterating the voluntary, anonymous nature of participation and the absence of any penalties or benefits for participating, was distributed to 1,800 employees across the hospital cluster. This pool included doctors, nursing staff, other healthcare professionals, and administrative and technical staff, all of whom had either permanent or fixed-term work relationships with the hospitals. Out of the 1,800 employees targeted, 363 individuals responded by successfully completing and submitting the questionnaire, representing a 20% response rate.

Ethics statement

The study received approval from the Ethics Committees of the Greek NHS hospital cluster in North Attica, encompassing the hospital units of Sismanoglio, Amalia Fleming, and Paidon Pentelis, under EC number 12813/14-6-18. The research was conducted following the principles of the 1964 Declaration of Helsinki and its later amendments.

Instrument of data collection

Since the purpose of this study was to assess the underlying constructs of motivation and job satisfaction of healthcare professionals (doctors, nurses, and administrative staff) a 27-item scale was used, as proposed by Labiris et al. (see Appendix). The first part of the instrument included 10 closed-ended questions for demographic and job-related characteristics (gender, age, educational level, marital status, hospital unit, hospital function, tenure, job position, work experience, and cyclical shift) [15]. The instrument’s second part included 27 items that measured 11 factors of motivation and job satisfaction, i.e., three items for achievement, one item for recognition, one item for advancement, two items for nature of work, one item for responsibility, five items for organization policy, two items for supervision, three items for job security, four items for interpersonal relations, and four items for salary satisfaction. Answers were reported in closed-ended questions in the form of a 5-point Likert scale (1=disagree strongly, 5=agree strongly). For this study, Cronbach’s alpha indices of derived factors of the 27-item instrument ranged from 0.811 to 0.913 indicating very good reliability.

Data analysis

Statistical analysis of the collected data was performed using IBM SPSS v.22 (IBM Corp., Armonk, NY). Demographic and job-related data were reported with absolute and relative frequencies (n, %). To address the first research question, i.e., identifying the drivers of employee satisfaction in the Greek NHS, exploratory factor analysis (EFA) was carried out on the 27-item job satisfaction questionnaire to identify the underlying factors. EFA involved assessing sample adequacy using the KMO measure and Bartlett’s test, using principal component analysis (PCA) for factor extraction, and retaining factors with eigenvalues greater than 1, followed by an appropriate rotation method to achieve a simpler and more interpretable factor structure. The three motivation and job satisfaction factors derived by EFA were also confirmed by confirmatory factor analysis (CFA), which evaluates the fit of the hypothesized factor structure. CFA criteria included a Comparative Fit Index (CFI) >0.9, Tucker-Lewis Index (TLI) >0.9, and Root Mean Square Error of Approximation (RMSEA) <0.08. CFA is a statistical technique used to verify the factor structure identified by EFA, ensuring that the data fits the proposed model well. The three derived factors were the dependent variables in further analysis of inferential statistics, with independent variables the job-related and demographic characteristics. These factors were described with mean and standard deviation values (M, SD) for the whole sample as well as for each group of employees. For the second research question, to detect possible differences between hospital units, functions, and job positions of employees, independent samples t-test, one-way analysis of variance (ANOVA), and three-way ANOVA were used. To examine the relationship between drivers of motivation and job satisfaction (third research question), cluster analysis was carried out using the two-step method to obtain employee profiles. The associations between demographics and job characteristics’ variables with job satisfaction and motivation profiles were reported via chi-square tests.

## Results

The sample of this study comprised 363 employees. Most participants were women (75.5%), in the age group of 46-55 years old (52.8%), employed as nursing staff (51.5%). Most employees were permanent staff (86.2%), while 11.8% were managers. The distribution of the sample in each hospital unit was balanced relative to each unit’s active employees as follows: Sismanoglio (54.8%), Amalia Fleming (24.2%), and Paidon Pentelis (20.9%). Detailed information about the sample’s characteristics is presented in Table 1.

**Table 1:** Demographics and job characteristics of the sample

Variable	Categories	Sample	Percentages (%)
Age groups (years)	18-25	1	0.3
	25-35	31	8.6
	36-45	94	26.0
	46-55	191	52.8
	56-77	45	12.4
Sex	Male	89	24.5
	Female	274	75.5
Hospital unit	Sismanoglio	199	54.8
	Amalia Flemig	88	24.2
	Paidon Pentelis	76	20.9
Hospital function	Medical	98	27.0
	Nursing	187	51.5
	Administration	78	21.5
	Technical	0	0.0
Tenure	Permanent	313	86.2
	Temporary	50	13.8
Job position	Manager	43	11.8
	Officer	320	88.2
Work experience groups (years)	0-2	49	13.5
	3-5	20	5.5
	6-10	50	13.8
	11-20	87	24.0
	20-40	157	43.3
Educational level	Master- PhD	79	21.8
	Bachelor	177	48.8
	High School	84	23.1
	Basic	23	6.3
Marital status	Married	257	70.8
	Single	69	19.0
	Widower	3	0.8
	Divorced	34	9.4
Cyclical shift	No	159	43.8
	Yes	204	56.2

EFA with Oblimin rotation was performed for the job satisfaction questionnaire (KMO 0.948, χ^2^(325)=5,288, p<0.000). Three factors emerged: 1) trust engagement (Cronbach’s alpha 0.913); 2) psychological contract (Cronbach’s alpha 0.811); 3) administrative model-professional relations-interactions (Cronbach’s alpha 0.895). Factor loadings and proposed structure, as well as the reliability indices, are presented in Table 2. CFA indicated a good fit of the data to the proposed factorial structure (χ^2^(282)=700.203, χ^2^/df=2.48, p<0.001, CFI 0.928, TLI 0.917, RMSEA 0.059). Descriptive statistics (M, SD) for the three factors are presented in Table 2. The factor of trust-engagement refers to the level of trust that the employees feel toward the administration and management team that serves the organizational goals to a good standard and pays attention to and respects employees’ needs and as a result there is an emotional connection with the leadership and hospital hierarchy. The factor of psychological contract represented the level of connection and positive feeling of the employees as public servants in a hospital where job stability, working environment, pension, etc., are secured and safeguarded by the state. The factor of administrative model and professional relations expressed the administrative framework in which they work; their interactions with their supervisors, peers, and colleagues; the level of communication; and teamwork cooperation in the daily working environment.

**Table 2:** Item loadings for the three factors solution of the job satisfaction questionnaire

Items	3-Factor solution item loadings		
	Trust-Engagement	Psychological contract	Administrative model and Professional relations
Q10	0.896	0.071	-0.16
Q20	0.848	-0.068	0.087
Q11	0.775	0.009	-0.057
Q19	0.705	-0.017	0.212
Q24	0.554	0.111	0.071
Q34	0.547	0.143	0.142
Q35	0.479	0.153	0.18
Q32	0.409	0.223	0.069
Q33	0.386	0.134	0.028
Q13	0.331	0.038	0.419
Q17	0.336	-0.033	0.373
Q12	0.349	0.222	0.166
Q28	0.005	0.706	0.003
Q26	0.144	0.655	-0.056
Q29	0.01	0.652	-0.064
Q30	0.039	0.634	0.032
Q27	0.063	0.583	0.022
Q18	0.314	-0.075	0.610
Q22	-0.149	0.069	0.882
Q23	0.08	-0.166	0.816
Q21	0.001	0.052	0.716
Q16	0.257	0.070	0.577
Q15	0.094	0.065	0.566
Q14	0.319	0.129	0.348
Q25	0.241	0.216	0.286
Q31	-0.045	0.247	0.258
Mean±SD	3.033 ± 0.854	2.782 ± 0.856	3.527 ± 0.942
Cronbach’s alpha	0.913	0.811	0.895

Independent sample t-tests were performed to examine possible differences between managers and employees in the three factors of job satisfaction (Table 3). Managers presented a higher level of trust-engagement (M=3.69, SD=0.792) compared to employees (M=2.94, SD=0.824), a difference which was statistically significant with a large effect size (d=0.928, t(361)=5.600, p<0.001). Furthermore, managers presented a higher level of psychological contract (M=3.19, SD=0.850) compared to employees (M=2.72, SD=0.840), a difference which was statistically significant with a medium effect size (d=0.556, t(361)=3.423, p=0.001). Managers presented a higher level of administrative model-professional relations (M=3.95, SD=0.780) compared to employees (M=3.47, SD=0.948), a difference which was statistically significant with a medium effect size (d=0.507, t(361)=5.600, p<0.001).

**Table 3:** Independent samples t-test results between managers and employees for the factors of job satisfaction M: mean value, SD: standard deviation, t-value: t-test statistic, P-value: statistical significance of the test. The significance level is 0.05.

Metric	Job position		Results	
	Manager (Mean±SD)	Employee (Mean±SD)	t-value	P-value
Trust-Engagement	3.69 ± 0.792	2.94 ± 0.824	5.600	<0.001
Psychological contract	3.19 ± 0.850	2.72 ± 0.840	3.423	0.001
Administrative model - Professional relations	3.95 ± 0.780	3.47 ± 0.948	3.172	0.002

One-way ANOVA indicated a statistically significant difference between the units of the hospital cluster for the managerial model-professional relations factor of job satisfaction (F(2,360)=4.536, p=0.011). More specifically (Table 4), the staff of Amalia Fleming presented higher levels of job satisfaction from administrative model-professional relations (M=3.79, SD=0.882) compared to Sismanoglio (M=3.45, SD=0.943) and Paidon Pentelis (M=3.42, SD=0.8963) hospitals, with a small to medium effect size (η^2^=0.025). Furthermore, there was a statistically significant difference between the functions of the hospital cluster for the psychological contract factor of job satisfaction (F(2,360)=4.976, p=0.007), indicating that the nursing staff had lower job satisfaction from psychological contract (M=2.65, SD=0.852) compared to medical (M=2.86, SD=0.774) and administrational staff (M=2.99, SD=0.918) with a small to medium effect size (η^2^=0.027).

**Table 4:** One-way ANOVA results for differences between hospital units and hospital functions for the factors of job satisfaction M: mean value, SD: standard deviation, ANOVA: analysis of variance, F-value: ANOVA statistic, P-value: statistical significance of the test. The significance level is 0.05.

Hospital units	Sismanogleio (Mean ± SD)	Amalia Fleming (Mean ± SD)	Paidon Pentelis (Mean ± SD)	F-value	P-value
Trust-Engagement	2.95 ± 0.885	3.13 ± 0.826	3.13 ± 0.791	1.904	0.150
Psychological contract	2.77 ± 0.877	2.81 ± 0.831	2.77 ± 0.837	0.058	0.943
Administrative model - Professional relations	3.45 ± 0.943	3.79 ± 0.882	3.42 ± 0.963	4.536	0.011
Hospital functions	Medical (Mean ± SD)	Nursing (Mean ± SD)	Administrational (Mean ± SD)			
Trust-Engagement	2.97 ± 0.895	3.02 ± 0.789	3.14 ± 0.949	0.880	0.416	
Psychological contract	2.86 ± 0.774	2.65 ± 0.852	2.99 ± 0.918	4.976	0.007	
Administrative model - Professional relations	3.48 ± 1.030	3.54 ± 0.878	3.56 ± 0.984	0.166	0.847	

A two-step cluster analysis was performed to examine possible employee profiles based on the levels of total job satisfaction, trust engagement, psychological contract, and administrative model. Three clusters were produced with an average silhouette score of 0.4 (Table 5), as follows: 1) highly satisfied employees (N=125), 2) somewhat satisfied employees (N=127), and 3) employees that are dissatisfied and unengaged (N=111).

**Table 5:** Motivation and job satisfaction profiles (clusters) and average levels of job satisfaction factors M: mean value, SD: standard deviation, F-value: ANOVA statistic, P-value: statistical significance of the test. The significance level is 0.05.

Measure	Cluster 1 (N=111) Unengaged-Dissatisfied	Cluster 2 (N=127) Somewhat satisfied	Cluster 3 (N=125) Highly Satisfied	F-value	P-value
	Mean±SD	Mean±SD	Mean±SD		
Job satisfaction (total)	2.347 ± 0.822	3.463 ± 0.619	4.336 ± 0.534	265.291	<0.001
Trust-Engagement	2.127 ± 0.480	2.955 ± 0.380	3.918 ± 0.497	460.305	<0.001
Psychological contract	2.059 ± 0.640	2.634 ± 0.512	3.574 ± 0.619	199.657	<0.001
Administrative model - Professional relations	2.474 ± 0.687	3.681 ± 0.525	4.306 ± 0.513	304.946	<0.001

As presented in Table 6, nursing staff had a lower prevalence in the cluster of highly satisfied employees (28.88%) compared to administrative-technical (42.31%) and medical staff (38.78%), indicating that nurses were less satisfied and motivated than other professionals. Work experience has a significant association with employee satisfaction profiles (χ^2^=16.112, p=0.041), with less experienced employees being more satisfied compared to more experienced employees. The managerial position presents the most significant association with employee profiles (χ^2^=20.981, p<0.001). Most managers are highly satisfied (65.12%), while most non-managerial staff are either dissatisfied-unengaged (33.13%) or somewhat satisfied (36.56%).

**Table 6:** Association of satisfaction profiles with demographic variables χ^2^: Chi-square statistic used to test the associations. P-value: p < 0.05 denotes statistical significance.

Category		Unengaged-Dissatisfied		Somewhat satisfied		Highly Satisfied		χ^2^	P-value
		N	%	N	%	N	%		
Hospital unit	Sismanogleio	70	35.18	69	34.67	60	30.15	5.974	0.201
	Amalia Fleming	20	22.73	33	37.50	35	39.77		
	Pedon Pentelis	21	27.63	25	32.89	30	39.47		
Gender	Female	87	31.75	101	36.86	86	31.39	4.642	0.098
	Male	24	26.97	26	29.21	39	43.82		
Age (years)	18-25	0	0.00	0	0.00	1	100.00	10.822	0.171
	25-35	9	29.03	15	48.39	7	22.58		
	36-45	25	26.60	37	39.36	32	34.04		
	46-55	66	34.55	62	32.46	63	32.98		
	56-77	11	24.44	12	26.67	22	48.89		
Education	MSc-PhD	21	26.58	27	34.18	31	39.24	5.393	0.494
	College	55	31.07	66	37.29	56	31.64		
	High School	31	36.90	25	29.76	28	33.33		
	Obligatory	4	17.39	9	39.13	10	43.48		
Hospital function	Medical	34	34.69	26	26.53	38	38.78	9.482	0.050
	Nursing	55	29.41	78	41.71	54	28.88		
	Administrative-Technical	22	28.21	23	29.49	33	42.31		
Work experience	0-2	10	20.41	14	28.57	25	51.02	16.112	0.041
	3-5	8	40.00	8	40.00	4	20.00		
	6-10	19	38.00	15	30.00	16	32.00		
	11-20	22	25.29	41	47.13	24	27.59		
	20-40	52	33.12	49	31.21	56	35.67		
Tenure	No	16	32.00	15	30.00	19	38.00	0.664	0.717
	Yes	95	30.35	112	35.78	106	33.87		
Manager	No	106	33.13	117	36.56	97	30.31	20.981	<0.001
	Yes	5	11.63	10	23.26	28	65.12		
Cyclical shift	No	43	27.04	50	31.45	66	41.51	6.281	0.043
	Yes	68	33.33	77	37.75	59	28.92		

## Discussion

This study aimed to explore Greek NHS employees’ views during the exit period from monetary and austerity measures (2018-2019), which conditions remained more or less up to nowadays where the lack of resources continues to affect the whole system negatively, in terms of the underlying factors that affect motivation and job satisfaction and possible differences in hospital units, hospital functions, and job positions, as well as the associated employee profiles and possible links of such profiles with demographic variables and job characteristics. Employees of the largest public hospital cluster in Attica, Greece, participated in this study, working in all hospital units and various hospital functions. After the outbreak of the financial crisis, several departments of this hospital cluster had been closed or absorbed and personnel were moved to other units. Moreover, Sismanoglio General Hospital had absorbed the human resources and equipment of a closed hospital unit in Attica, and Amalia Fleming had been downsized in facilities and human resources. From the perspective of corporate governance, the three hospital units remained separate, acting as independent hospitals without a common organogram but only a common administration board, CEO and three deputy managers who were all assigned by the Ministry of Health. There were overlapping and low economies of scale, a different set of priorities, and very limited employee mobility. A budget decrease of almost 50% was implemented between 2012 and 2018 with demand for the provision of services remaining unchanged and the personnel income shrinking by half compared to the pre-crisis period. It was therefore of great interest to address the drivers of motivation and job satisfaction for the employees after undergoing such severe organizational changes.

Three factors emerged from the exploratory analysis of the motivation and job-satisfaction questionnaire of this study: trust engagement, psychological contract, and administrative model-professional relations. The derived factors that indicate the drivers of motivation and job satisfaction in the Greek NHS, agreed with the three distinguished factors of ERG theory, i.e., existence needs, relatedness needs, and growth needs. More specifically, existence needs are related to salary, future pensions, safety, job security, healthcare services, etc., which describe the working conditions of a public servant in the healthcare sector and are directly related to the psychological contract factor of this study. Furthermore, relatedness needs in the hospital environment include the interactions and relationships that develop between employees and are affected by the administrative and regulatory framework, the hierarchy model, rules, and employee code of ethics, which are all part of the administrative model-professional relations factor that emerged in this study. It is worth mentioning that in the complex environment of a public hospital [16-18], teamwork plays a significant role in the workflow process. This is due to employees working in multidisciplinary teams, including nurses, doctors, and administrative staff of different specialties. To accomplish their tasks, these employees must work effectively in a team, communicating and sharing resources; thus, relatedness needs play a critical role in employees’ motivation when positive professional relations and interactions exist in the hospital environment. Moreover, previous research has shown that individual and team motivation [6], quality of professional relationships [8], and psychological contract strength [9,10] are critical factors for the level of employee and organizational performance, especially when available resources are limited [6].

The third ERG factor of growth needs refers to self-esteem and self-fulfillment of employees, i.e., the need to be useful and productive in the workplace and is thus related to the trust-engagement factor that emerged in this study. Individual’s growth needs within an organization are fulfilled when the employees are engaged and aligned with the goals and mission of the organization, as work engagement is a positive, fulfilling state of mind, an affective-motivational state of high energy, dedication, and strong focus on work that leads to creativity, task performance, and organizational citizenship behavior [12,19,20]. The trust-engagement factor of this study also refers to the commitment of employees to the organization, a positive work-related state that is based more on trust and inspiration than specific professional growth paths or monetary rewards and bonuses and has been shown to enhance organizational performance [7,21,22].

Based on the results of this study, ERG theory may offer a good framework to examine healthcare employees’ motivation and job satisfaction, especially when there is evidence that Hertzberg’s model of retention/hygiene and motivation sub-scales presents limitations [23]. In complex multidisciplinary settings, in particular, such as hospital environments where job satisfaction is derived not only from static job characteristics and conditions but also from the interactions with co-workers and patients, as well as the processes guiding day-to-day tasks, the underlying constructs of motivation and job satisfaction could be examined in terms of a more content- and process-based theory, such as Alderfer’s ERG theory [14].

Furthermore, this study showed that despite the severe psychological contract breach, other motivation, and job satisfaction factors such as trust, engagement, and work relationships, were high, even though employees faced three memorandums, austerity measures, and partial downsizing of the hospital cluster. Other studies of healthcare professionals at public hospitals in Attica and Northern Greece, as well as in other countries, have found that work connections [16,24,25] are a key motivator in public hospital settings. Moreover, satisfaction with work conditions and supervisor support had a higher impact on the job satisfaction of nurses [26]. These results make it clear that, for the hospital's management to be able to improve the level of employee performance, it should ensure the establishment of a strong climate among employees, and also acknowledge the efforts made by them. Realizing that in a public hospital, motivation factors are the trustful relations across teams, appreciation demonstrated by managers and colleagues, job security, training, stable income, and trust and engagement with the leadership team, while the main discouraging parameters are low annual income, high taxes, limited resources, budget cuts, and burnout, will lead to HR policies with targeted strategies to increase staff commitment and ultimately improve performance [27-29].

Managers presented higher levels of motivation and job satisfaction compared to other hospital employees. Job seniority and position have been related to increased job satisfaction in nurses before the COVID-19 pandemic. This difference between employees and managers might be explained through locus of control theory [29] or the fact that managers presented higher situational awareness compared to non-managers [20,30]. Yet, the Greek NHS administrative model could be characterized as bureaucratic and authoritative with very limited flexibility and mobility [24]. Most of the managers in all functions, but mainly in nursing, administrative, and technical staff, were assessed and assigned through an official process at least 10 years ago or positioned by the hospital CEO, through an internal process. Occasionally, these assignments were influenced by political criteria rather than crucial or typical managerial skills. Moreover, the annual typical performance appraisal that each manager conducts of their subordinates is not linked to business plans, operations, or HR targets. This leads often to “permanently” occupied positions without succession plans or turnover according to the accomplishment of targets, concluding that managers feel more secure occupying these “permanent” positions, more satisfied and engaged with the system compared to the other employees [30].

The nursing staff in this study presented lower job satisfaction compared to medical and administrative staff, as has been reported also by a previous Greek study [17]. It should be mentioned that Greece has one of the lowest ratios of nurses per 1,000 population (3.3) and one of the highest ratios of doctors per 1,000 population (6.6) compared to the average ratios of the Organization for Economic Co-operation and Development (OECD) countries (8.7 for nurses and 3.4 for doctors). Therefore, low satisfaction levels may be explained by increased workload, limited resources, and the higher levels of burnout that they suffer [26].

Limitations

This study is among the first in Greece to explore motivation and job satisfaction factors among Greek NHS employees in relation to the ERG theory during the period of partial recovery from the financial crisis, within the context of severe financial constraints imposed during the crisis. However, it has certain limitations. Firstly, the cross-sectional design does not allow for the identification of causal relationships. Secondly, the study's instrument, a questionnaire based on Hertzberg’s motivation theory, limits the assessment of Alderfer’s “growth needs.” Growth needs were examined indirectly through the trust-engagement derived factor, but future research could address the actual dimensions of growth needs specific to the complexity of the hospital environment. Thirdly, the study's sample was restricted to employees of a single hospital cluster serving a specific geographical area in Attica, Greece’s largest prefecture. Additionally, another limitation was the lack of control over confounding variables such as the quality of life and financial resources of professionals. Lastly, due to the nature of the sampling procedure, sampling bias may be a concern as only employees willing to participate completed the questionnaires.

## Conclusions

This study provided significant insights into the motivation and job satisfaction of Greek NHS employees during a period of financial constraint, highlighting intrinsic factors such as professional relationships and trust as critical drivers of motivation and satisfaction. Despite the severe psychological contract breach after the resource constraints imposed due to austerity measures, these factors remained influential. Managers in all hospital functions reported higher levels of job satisfaction while the nursing staff presented lower levels of motivation and satisfaction highlighting the need for further addressing the low ratio of nurses in Greece compared to doctors per 1,000 population. These data provide a baseline against which future research may compare the motivation and job satisfaction levels of Greek NHS employees, especially during the post-COVID-19 pandemic period and before the implementation of the recent reforms that the government decided to semi-privatize the Greek NHS system. The evidence provided may also inform Greek NHS management about the state of employees’ behavioral interactions, performance potential, and readiness to manage effectively not only day-to-day tasks but also public health crises such as the ongoing COVID-19 pandemic that has burdened the Greek NHS since the first quarter of 2020.

## Appendices

**Table 7:** Job Satisfaction Questionnaire for Greek Mental NHS Hospitals Developed by Labiris et al. [15] and modified by Koutalas E.

Job Satisfaction Questionnaire	
Part 1: Sociodemographic and Job-Related Information	
Question	Response
Age: (Please specify your age)	
Gender: (Male, Female)	
Education Level: (Secondary Education, Bachelor's Degree, Master's Degree, Doctorate, Other)	
Position: (Please specify your job title)	
Years of Experience: (Please specify the number of years you have been working in your current position)	
Marital Status: (Single, Married, Divorced, Widowed)	
Department: (Please specify the department you work in)	
Employment Type: (Full-time, Part-time, Contractual, Temporary)	
Part 2: Job Satisfaction and Motivational Factors (Using a 5-point Likert scale: 1 = Strongly Disagree, 5 = Strongly Agree)	
Section	Question
Trust–Engagement	
Q10	I trust the management to meet the goals of the hospital.
Q20	I feel that I can trust my management/directorate because they make good decisions.
Q11	I am informed about the progress and goals of the hospital and am given opportunities to participate in the implementation of the goals.
Q19	I feel that I can trust the management/administration and that they support me.
Q24	The training provided to me by the Hospital is appropriate and adequate.
Q34	Management rewards me for the quality of my work.
Q35	I feel proud to work at the Sismanoglio Hospital.
Q32	There is satisfactory cooperation between the Divisions/Units/Departments of the Hospital.
Q33	Social events are organised during the year to develop interpersonal relationships (e.g. holiday season).
Q13	I feel part of a team that has common goals.
Q17	I feel that I am useful to the hospital, I use my skills and contribute with my work.
Q12	My working conditions (premises, cleanliness, equipment, etc.) are good.
Psychological Contract	
Q28	The health care provided to me is satisfactory.
Q26	The remuneration of my work is satisfactory for my qualifications and duties.
Q29	The current retirement scheme from the Hospital is satisfactory.
Q30	I feel secure with my employment status.
Q27	The leaves granted (regular, educational, personal, etc.) are satisfactory.
Administrative Model and Professional Relations	
Q18	I feel that I can trust my supervisors because they make good decisions.
Q22	My supervisors treat me with respect.
Q23	My supervisors take my opinion into account in making decisions about changes that are made that affect my work activity in the Team/Department/Unit, etc.
Q21	My supervisors tell me when my work needs improvement and when I am doing it right (support and guidance from my immediate supervisor).
Q16	When my work is good it is recognized by management/ Directorate (appreciation and good treatment by the supervisor).
Q15	There is cooperation and communication in the unit/department where I work.
Q14	I like the work I do, and the tasks assigned to me are defined.
Q25	The quality of the services provided is a priority in the place where I work.
Q31	I am mainly interested in the results of my work rather than formalism.

## References

[1] Kentikelenis A, Karanikolos M, Reeves A (2014). Greece’s health crisis: from austerity to denialism. Lancet.

[2] Simou E, Koutsogeorgou E (2014). Effects of the economic crisis on health and healthcare in Greece in the literature from 2009 to 2013: a systematic review. Health Policy.

[3] Goldsmith SB (2005). Principles of Health Care Management: Compliance Consumerism and Accountability in the 21st Cen. http://books.google.gr/books?hl=en&lr=&id=A7KxaxkG2rMC&oi=fnd&pg=PR11&dq=Principles+of+Health+Care+Management:+Compliance+Consumerism+and+Accountability+in+the+21st+Cen&ots=IHeADbpVJk&sig=JRFRhokWR0Js_1K3ucz3HgGvRfw&redir_esc=y#v=onepage&q=Principles%20of%20Health%20Care%20Management%3A%20Compliance%20Consumerism%20and%20Accountability%20in%20the%2021st%20Cen&f=false.

[4] Kerasidou A, Kingori P, Legido-Quigley H (2016). "You have to keep fighting": maintaining healthcare services and professionalism on the frontline of austerity in Greece. Int J Equity Health.

[5] (2024). People at risk of poverty or social exclusion. https://ec.europa.eu/eurostat/web/products-eurostat-news/w/ddn-20230614-1.

[6] Rachiotis G, Kourousis C, Kamilaraki M, Symvoulakis EK, Dounias G, Hadjichristodoulou C (2014). Medical supplies shortages and burnout among Greek health care workers during economic crisis: a pilot study. Int J Med Sci.

[7] Sbarouni V, Tsimtsiou Z, Symvoulakis E (2012). Perceptions of primary care professionals on quality of services in rural Greece: a qualitative study. Rural Remote Health.

[8] Simou E, Karamagioli E, Roumeliotou A (2015). Reinventing primary health care in the Greece of austerity: the role of health-care workers. Prim Health Care Res Dev.

[9] Robinson SL, Rousseau DM (1994). Violating the pscyhological contract: not the exception but the norm. J Organisational Behav.

[10] Raja U, Johns G, Ntalinais F (2004). The impact of personality on psychological contracts. Academy Manag J.

[11] Koutalas E, Kostares E, Paraskevadaki E (2024). Scenarios for assessing leadership behaviors of mid-level managers in the Greek national health system through the lens of servant leadership theory: a pilot study in the hospital cluster of North Attica. Cureus.

[12] Herzberg F (1968). One more time: how do you motivate employee?. Harv Bus Rev.

[13] Markovits Y, Davis AJ, Fay D (2010). The link between job satisfaction and organizational commitment differences between public and private sector employees. Int Public Manag J.

[14] Alderfer CP (1969). An empirical test of a new theory of human needs. Organizational Behavior and Human Performance. Organ Behav Human Perform.

[15] Labiris G, Gitona K, Drosou V, Niakas D (2008). A proposed instrument for the assessment of job satisfaction in Greek mental NHS hospitals. J Med Syst.

[16] Lambrou P, Kontodimopoulos N, Niakas D (2010). Motivation and job satisfaction among medical and nursing staff in a Cyprus public general hospital. Hum Resour Health.

[17] Gaki E, Kontodimopoulos N, Niakas D (2013). Investigating demographic, work-related and job satisfaction variables as predictors of motivation in Greek nurses. J Nurs Manag.

[18] Krogstad U, Hofoss D, Veenstra M, Hjortdahl P (2006). Predictors of job satisfaction among doctors, nurses and auxiliaries in Norwegian hospitals: relevance for micro unit culture. Hum Resour Health.

[19] Schaufeli WB, Salanova M, González-Roma V (2002). The measurement of engagement and burnout: a two-sample confirmatory factor analytic approach. J Happiness Studies.

[20] Bakker AB, Albrecht S (2018). Work engagement: current trends. Career Develop Int.

[21] Bohm J (2012). Two-factor theory - at the intersection of health care management and patient satisfaction. Clinicoecon Outcomes Res.

[22] Vandenabeele W (2007). Toward a public administration theory of public service motivation. Public Manag Rev.

[23] Markovits Y, Davis AJ, Dick R (2007). Organizational commitment profiles and job satisfaction among Greek private and public sector employees. Int J Cross Cultural Manag.

[24] Aletras V, Kontodimopoulos N, Zagouldoudis A, Niakas D (2007). The short-term effect on technical and scale efficiency of establishing regional health systems and general management in Greek NHS hospitals. Health Policy.

[25] Kitsios F, Kamariotou M (2021). Job satisfaction behind motivation: an empirical study in public health workers. Heliyon.

[26] Liu C, Zhou H, Jin Y, Chuang YC, Chien CW, Tung TH (2022). Application of a hybrid multi-criterion decision-making model for evaluation and improvement of nurses’ job satisfaction. Front Public Health.

[27] Kalinowska P, Marcinowicz L (2020). Job satisfaction among family nurses in Poland: a questionnaire-based study. Nurs Open.

[28] Nurmeksela A, Mikkonen S, Kinnunen J, Kvist T (2021). Relationships between nurse managers' work activities, nurses' job satisfaction, patient satisfaction, and medication errors at the unit level: a correlational study. BMC Health Serv Res.

[29] Padmanabhan S (2021). The impact of locus of control on workplace stress and job satisfaction: a pilot study on private-sector employees. Current Res Behav Sci.

[30] Martin SL, Klimoski RJ, Nicolaides V (2019). Situation awareness in management: making the implicit explicit. Academy Manag Proc.

